# The 5’-AMP-Activated Protein Kinase (AMPK) Is Involved in the Augmentation of Antioxidant Defenses in Cryopreserved Chicken Sperm

**DOI:** 10.1371/journal.pone.0134420

**Published:** 2015-07-29

**Authors:** Thi Mong Diep Nguyen, François Seigneurin, Pascal Froment, Yves Combarnous, Elisabeth Blesbois

**Affiliations:** 1 INRA-CNRS, Unité Mixte de Recherche de Physiologie de la Reproduction et des Comportements, F-37380 Nouzilly, France; 2 Université François Rabelais, Tours, France; 3 SYSAAF, F-37380 Nouzilly, France; Clermont-Ferrand Univ., FRANCE

## Abstract

Semen cryopreservation is a unique tool for the management of animal genetic diversity. However, the freeze-thaw process causes biochemical and physical alterations which make difficult the restoration of sperm energy-dependent functions needed for fertilization. 5’-AMP activated protein kinase (AMPK) is a key sensor and regulator of intracellular energy metabolism. Mitochondria functions are known to be severely affected during sperm cryopreservation with deleterious oxidative and peroxidative effects leading to cell integrity and functions damages. The aim of this study was thus to examine the role of AMPK on the peroxidation/antioxidant enzymes defense system in frozen-thawed sperm and its consequences on sperm functions. Chicken semen was diluted in media supplemented with or without AMPK activators (AICAR or Metformin [MET]) or inhibitor (Compound C [CC]) and then cryopreserved. AMPKα phosphorylation, antioxidant enzymes activities, mitochondrial potential, ATP, citrate, viability, acrosome reaction ability (AR) and various motility parameters were negatively affected by the freeze-thaw process while reactive oxygen species (ROS) production, lipid peroxidation (LPO) and lactate concentration were dramatically increased. AICAR partially restored superoxide dismutase (SOD), Glutathione Peroxidase (GPx) and Glutathione Reductase (GR), increased ATP, citrate, and lactate concentration and subsequently decreased the ROS and LPO (malondialdehyde) in frozen-thawed semen. Motility parameters were increased (i.e., + 23% for motility, + 34% for rapid sperm) as well as AR (+ 100%). MET had similar effects as AICAR except that catalase activity was restored and that ATP and mitochondrial potential were further decreased. CC showed effects opposite to AICAR on SOD, ROS, LPO and AR and motility parameters. Taken together, our results strongly suggest that, upon freeze-thaw process, AMPK stimulated intracellular anti-oxidative defense enzymes through ATP regulation, thus reducing ROS and lipid peroxidation, and consequently partially restoring several essential sperm functions and leading to a better quality of cryopreserved sperm.

## Introduction

Semen cryopreservation is a key tool to manage the conservation of animals genetic diversity. This process is highly successful in many mammalian species, but is still difficult in birds due to their specific adaptive reproductive process that enhances their need for a highly efficient energetic supply and maintenance of sperm functions [1–3].

Semen cryopreservation leads to the death of a significant proportion of sperm in all species (40–60% in the chicken), and to the alteration of many functions of the surviving sperm. Different aspects of the energetic metabolism have been reported to be affected by sperm cryopreservation with consequences on motility regulation, sperm membrane integrity and ATP content in mammals [4, 5] as well as in birds [6].

AMPK is a key sensor and regulating kinase of energetic metabolism. Its numerous roles include regulation of glucose, lipid, and protein metabolisms. AMPK is a heterotrimeric protein consisting of a catalytic α-subunit and two regulatory subunits, β and γ, with different species and tissue-specific isoforms. Phosphorylation of a specific threonine residue (Thr172) of the α-subunit is crucial for AMPK activity that switches cells from an anabolic to a catabolic state, shutting down the ATP-consuming synthetic pathways and restoring energy balance [7–9]. Recently, AMPK activation has been reported to increase the expression of antioxidant enzymes in monocytes macrophages [10], restore glutathione (GSH) depletion and reduce reactive oxygen species (ROS) levels in rat diabetic fibrosis, kidney tissues and humans [11–13], suppress ROS production in bovine endothelial cells [14] and increase anti-inflammatory reactions in mice leucocytes [15]. However, the AMPK antioxidants stimulating effect on mature sperm properties or their cryopreservation have never been reported.

Because sperm membranes are enriched in polyunsatured fatty acids (PUFAs) in mammalian [16] and bird species [17], sperm are very susceptible to lipid peroxidation (LPO) with subsequent alterations of structure and functions [18, 19]. Superoxide dismutase (SOD), glutathione peroxidase (GPx), glutathione reductase (GR) and catalase (CAT) are the major antioxidant enzymes naturally present in mammalian and bird semen to protect sperm from lipid peroxidation and to maintain its integrity against ROS [20–22]. Freeze-thaw process have been shown to induce significant reduction in human sperm SOD [23] and in bovine sperm antioxidant defenses [24] with increases in superoxide anions (O_2_^•−^) and hydrogen peroxide (H_2_O_2_) concentrations as well as inhibitions of both ATP production and sperm movement, particularly forward progression [25]. Reductions in SOD, GPx, Catalase activities, and increases in ROS and LPO have been shown after chicken sperm cryopreservation [26], but these observations have never been connected to AMPK regulation. The aim of this study was to examine the role of AMPK on the peroxidation/antioxidant defense enzymes system of frozen-thawed sperm and its effects on sperm functions. Chicken semen was diluted in media supplemented or not with AMPK activators (AICAR or MET) or inhibitor (Compound C) and then cryopreserved. AMPK phosphorylation, antioxidant enzymes activities, peroxidation, mitochondrial, energetic indicators, and sperm functions (motility and ability to perform acrosome reaction) were also evaluated before and after cryopreservation.

## Materials and Methods

### Chemicals and reagents

All chemicals were purchased from Sigma–Aldrich (St Louis, MO, USA) unless otherwise noted. Compound C (CC) also named Dorsomorphin: 6-[4-(2-Piperidin-1-yl-ethoxy)-phenyl]-3-pyridin-4-yl-pyrazolo[1,5-a]pyrimidine, AICAR: 5-aminoimidazole-4-carboxamide-1-β-d-ribofuranoside (AICAR), and Metformin (MET): 1,1-dimethylbiguanide hydrochloride were obtained from Calbiochem (Billerica, MA). A stock solution of CC was prepared in dimethylsulphoxide (DMSO) and stock solutions of MET and AICAR were prepared in deionized water. Complete mini EDTA-free, protease inhibitor cocktail tablets were from Roche diagnostics (Mannheim, Germany). Tris/glycine buffer (10X), Tris/glycine/SDS buffer (10X), and Precision Plus Protein All Blue Standards (Catalog #161–0373) were obtained from Bio-Rad (Hercules, CA) and anti-AMPKα from Millipore (Billerica, MA), anti-phospho-Thr^172^-AMPKα and anti-rabbit IgG (H+L) (DyLight 680 Conjugate) antibodies from Cell Signaling technology, Inc (Danvers, MA). SYBR-14/PI (LIVE/DEAD sperm viability kit) and JC1 were purchased from Molecular Probes (Saint Aubin, France). The LPO-586 kit was from Oxis Research (Burlingame, CA, US).

### Animals

All experiments were carried out in accordance with the legislation governing the ethical treatment of animals and approved by the Ethics Committee (“Comité d'Éthique en Expérimentation Animale du Val de Loire”, Tours, France, N° 19).

The males were 50–65 week-old adult chickens of the meat type D+/- lines [27]. All the animals were housed in individual battery cages under a 14L/10D photoperiod and fed with a standard diet of 12.5 MJ/day.

### Semen collection

Semen was routinely collected twice a week by the abdominal massage method [28]. Using this technique does not harm the animals: they are simply caught by hand and free to go after the abdominal massage, without suffering any injury. Sperm concentration was determined by light absorption of semen at 530nm with a photometer (IMV, L’Aigle, France). The semen from fifteen different males were gently mixed together after collection and split into four samples further divided according to the number of treatments. Fresh sperm were diluted in Beltsville Poultry Semen Extender (BPSE) [29] to get a final sperm concentration of 1 x 10^9^ cells/ml before further use. BPSE buffer contains 2mM potassium citrate, 45mM sodium glutamate, 1.7mM magnesium chloride, 5mM K_2_HPO_4_, 4.7mM KH_2_PO_4_, 16mM TES, 32mM sodium acetate and 27.8mM glucose; pH 7.3 and osmolality 350 mOsmol/kg.

### Sperm cryopreservation

The semen for freezing was diluted 1:1 with Lake PC [30] (composition: 5.6mM magnesium acetate, 113.5mM sodium glutamate, 50.9mM potassium acetate, 44.4mM D-fructose, 3g polyvinylpyrrolidone; adjusted to 1L with distilled water; adjusted to pH 7.1 with NaOH and osmolality 340mOsmol/kg) in the presence or absence of 5μM CC, or 2mM AICAR, or 1mM MET. At the same time, a second solution of Lake PC with 11% of glycerol cryoprotectant was prepared. They were both cooled separately at 4°C for 10min and then mixed together and equilibrated for another 10min at 4°C [31]. After equilibration, the semen was transferred to 0.5ml plastic freezing straws (IMV, L’Aigle, France) which were sealed and finally frozen from +4 to -35°C at -7°C/min and from -35 to -140°C at -60°C/min using a programmable Minidigitcool 1400 freezer (IMV, L’Aigle, France). The straws were then plunged into liquid nitrogen (-196°C). Six cryopreservations (each cryopreservation including four different samples/treatment) were done.

### Thawing Procedures

Sperm was thawed for 4min in a water bath adjusted to 4°C. After thawing, the straws were quickly opened and semen transferred to a glass beaker. Semen was progressively diluted (6 times 2min) with Lake Centri buffer [32] (composition: 5.6mM magnesium acetate, 113.5mM sodium glutamate, 13mM potassium acetate, 44.4mM D-fructose, 62.2mM sodium acetate; adjusted to 1L with distilled water; pH 7.1 and osmolality 350mOsmol/kg) at 4°C to final dilution of 1:19. Glycerol was removed by centrifugation (15min at 700G, 4°C) [31]. After removal of the supernatants, the resulting pellets were resuspended in 100ml of Lake 7.1 [33] (composition: 5.6mM magnesium acetate, 4.2mM tripotassium citrate, 89.9mM sodium glutamate, 44.4mM glucose, 143mM Bes (N, N-bis[2-hydroxyethyl]-2-aminoethanesulfonic acid), 4% NaOH, adjusted to 1L with distilled water; pH 7.1 and osmolality 370mOsmol/kg). Concentration of sperm was estimated at a wavelength of 530nm. Concentrations were close to 1 x 10^9^ cells/ml.

### Sperm viability assessment

SYBR-14/PI was used to assess sperm viability [34] before freezing and after thawing. Aliquots from each sample were adjusted to a final concentration of 1 x 10^9^ cells/ml. Then, sperm were diluted in Lake 7.1 buffer down to 20 x 10^6^ cells/ml and 5µl SYBR-14 was added before the solution was incubated for 10min in darkness at 4°C. Afterwards, 2µl of propidium iodide (PI) was added and the incubation was continued for 5min in the dark at 4°C. After incubation, sperm viability was assessed by fluorescence microscopy (Zeiss Axioplan 2; Zeiss Gruppe, Jena, Germany): living cells appeared green and dead ones red. A total of 300 sperm per slide were counted (two slides/sample = 1 replicate) and a total of six replicates/treatment examined. All preparations were analyzed by the same observer.

### Analysis of sperm motility by computer-assisted sperm analysis (CASA) system

The sperm motility parameters were evaluated by the computer-assisted sperm analysis (CASA) system with an HTM-IVOS (Hamilton-Thorn Motility Analyzer, IVOS) [35]. In this experiment, the parameters measured were percentage of motile sperm (%), curvilinear velocity (VCL, in μm/s), path velocity (VAP, in μm/s), progressive velocity (VSL, in μm/s), straightness coefficient (STR, in %) (STR = VSL/VAP*100), linearity (LIN, in %) (LIN = VSL/VCL*100). Following these parameters, percentage of motile sperm was defined as the percentage of spermatozoa showing a VAP > 5μm/s, progressive cells were defined as sperm having VAP > 50μm/s and STR > 75%, rapid cells were defined as having VAP > 50μm/s, slow cells were defined as having VAP < 50μm/s, and static cells (sperm not moving at all) were defined as having VAP ≤ 5μm/s.

### Acrosome reaction assessment with FITC-PNA

The acrosome reaction (AR) completion was detected by FITC-conjugated peanut agglutinin (FITC-PNA) binding [36]. Sperm were incubated at 41°C with 50μl of the inner perivitelline layer (IPVL) and 500μl of NaCl-TES (TES: N-Tris-[hydroxymethyl]-methyl-2-aminoethanesulfonic acid) containing 5mM Ca^2+^ for 5min. The samples were then centrifuged at 400g for 5min and the pellets resuspended in 100μl NaCl-TES. FITC-PNA was then added (1mg/ml) and the sperm were incubated for 10min in the dark at 4°C, then washed in 440μl of NaCl-TES and centrifuged at 400g for 5min. The pellets were resuspended in 200μl NaCl-TES for analysis. The sperm that have completed their AR were observed under fluorescence microscopy (Zeiss Axioplan 2; Zeiss Gruppe, Jena, Germany). A minimum of 100 sperm were counted for each sample (two slides/sample = 1 replicate) and a total of six replicates/treatment examined. Sperm having completing their AR were characterized by the green fluorescence of the acrosomal region [37, 38]. All preparations were analyzed by the same observer.

### Assessment of sperm mitochondrial membrane potential

The activity of mitochondria within the sperm midpiece was determined using the mitochondrial probe 5,5’,6,6’-tetra-chloro-1,1’,3,3’-tetraethylbenzimidazolyl-carbocyanine iodide (JC-1; Invitrogen, Cergy-Pontoise, France). Sperm were combined with 2μM JC-1 and incubated for 20min at 37°C. They were examined using standard immunofluorescence technique. Sperm mitochondria with bright orange fluorescence at the midpiece region were considered to be positive (high membrane potential) for mitochondrial activity, whereas those exhibiting a green fluorescence were categorized as having low mitochondrial membrane potential. The percentage of orange stained cells was recorded and considered as the population of sperm with a high mitochondrial membrane potential. A total of 300 sperm per slide were counted (two slides/sample = 1 replicate) under a fluorescence microscope (Zeiss Axioplan 2; Zeiss Gruppe, Jena, Germany) and a total of six replicates/treatment examined. All preparations were analyzed by the same observer. Results are expressed as the average of the percentage of orange stained sperm ± SEM.

### Adenosine triphosphate (ATP) concentration measurement

After incubation of sperm with or without AMPK modulators, ATP concentration in sperm was measured using the luciferin/luciferase reactions with Cell-Titer-Glo Assay (Promega, Madison, WI, USA). Standards were prepared from ATP standard (F203A, Promega) using serial dilutions to obtain concentrations of 1×10^−7^, 1×10^−8^, 1×10^−9^, 1×10^−10^, 1×10^−11^ and 1×10^-12^M. Briefly, the assay buffer and substrate were equilibrated to room temperature, and the buffer was transferred to the substrate and gently mixed with it to obtain a homogeneous solution. After a 30min equilibration of the cell plate to room temperature, addition of 100μl sample and 100μl luciferin/luciferase reagent was carried out in 96-well plates, the content was mixed for 2min and incubation was continued for 10min at room temperature. The luminescence at integration time 1000 (ms) was read using an Ascent Luminoskan Luminometer (Thermo) with Lake 7.1 [33] as a blank for each experiment.

### Determination of ROS production

A modified colorimetric Nitro Blue Tetrazolium (NBT) (Sigma–Aldrich) test was used to evaluate sperm ROS production [39, 40]. After having been washed in phosphate buffer (pH 7.0), sperm were resuspended in 200μl of phosphate buffer and incubated with an equal volume of NBT reagent (1mg/ml) at 37°C for 45min. Following incubation, the samples were washed and centrifuged twice at 500g for 10min in phosphate buffer to remove all residual NBT solution, leaving only a cell pellet containing formazan. For quantification, the intracellular formazan was solubilized in 60μl of 2M KOH and DMSO and the absorbance at 630nm was determined. A standard curve was prepared by adding KOH and DMSO to known amounts of NBT. ROS production was expressed as μg of formazan/2.10^8^ sperm.

### Determination of lipid peroxidation (LPO)

The level of LPO in sperm was determined using commercial kit LPO-586 (Oxis Research, Burlingame, CA, US). The method is based on the reaction of a chromogenic reagent, N-methyl-2-phenylindole (R1) with malondialdehyde (MDA) and 4-hydroxyalkenals at 45°C. The colorimetric reaction in 200μl of the supernatant was produced by the addition of 650μl of 10.3mM N-methyl-2phenyl-indole diluted to a 3:1 acetonitrile:methanol mixture. The reaction was started by the addition of 150μl of methanesulfonic acid. The mixture was strongly vortexed and incubated at 45°C for 1h and then centrifuged at 3000rpm for 10min. The absorbance in the supernatant was read at 586nm (Bio-Rad spectrophotometer; Hercules, CA, USA). The absorbance values were compared to a standard curve in the concentration range of 0.5 to 4μM of 1,1,3,3-tetramethoxypropane (10mM stock) to calculate the malonyldialdehyde and 4-hydroxyalkanal contents in the samples.

### Determination of superoxide dismutases (SOD)

SOD are a group of metalloenzymes that detoxify ROS through the conversion of O_2_^•−^ to H_2_O_2_ and molecular oxygen. Total SOD activity was assessed by a spectrophotometric method based on the inhibition of a superoxide-induced epinephrine oxidation [41]. O_2_^•−^serves as chain propagation species for the autoxidation of epinephrine to adrenochrome. Since SOD competes with this reaction, it slows down adrenochrome formation. After having been washed in phosphate buffer (pH 7.0), 200μl of semen sample were centrifuged at 800g at 4°C for 10min, the supernatants were then collected and 0.05M carbonate buffer (pH 10.2) containing 0.1mM EDTA followed by 30mM epinephrine in 0.05% v/v acetic acid were added. The change in SOD activity was measured at 480nm for 4min. SOD production was expressed as U/2.10^8^ sperm. For control, samples were replaced by carbonate buffer, pH 10.2.

### Determination of catalase activity (CAT)

CAT activity was evaluated by the method of Goth (1986) [42], which is based on H_2_O_2_ degradation by the action of CAT contained in the examined samples. The reaction mixture consisted of 50mM potassium phosphate (pH 7.0), 2μl of 95% ethanol, and 200μl of semen sample incubated at 4°C for 30min. Then 2μl of 1% Triton 100 were added and incubation was performed at 4°C for 10min. The reaction was initiated by the addition of 30mM of H_2_O_2_, and absorbance changes were measured at 405nm. The CAT activity was expressed as U/2.10^8^ sperm.

### Determination of glutathione peroxidase activity (GPx)

The GPx activity was determined using the method described by Lawrence and Burk [43]. GPx catalyzes the reaction between H_2_O_2_ and reduces glutathione (GSH) to form oxidized glutathione (GSSG) and water utilizing nicotinamide adenine dinucleotide phosphate (NADPH). The oxidation of NADPH to NADP^+^ was accompanied by a decrease in absorbance at 340nm providing a spectrophotometric means for monitoring GPx activity. The GPx activity in sperm was expressed as mU/2.10^8^ sperm using the extinction coefficient of NADPH as 6.22. 200μl of semen sample were added with 0.8ml of 50mM potassium phosphate buffer (pH 7.0), 1mM EDTA, 1mM sodium azide, 0.2mM NADPH, 100μl of oxidized glutathione reductase, 1mM GSH, and 0.25mM H_2_O_2_, and was incubated at 30°C for 5min.

### Determination of Glutathione Reductase (GR)

Glutathione reductase (GR) catalyses the reduction of oxidized glutathione (GSSG) by NADPH to GSH. The activity of GR in sperm was measured by the method of Pinto and Bartley (1969) [44]. 200μl of semen sample were added with 0.5ml of 0.25M potassium phosphate buffer (pH 7.0), 1ml of 25mM EDTA, 0.1ml of 1mM NADPH, and 1ml of distilled water. The reaction was initiated by the addition of 50mM of GSSG. The activity was expressed as mU/2.10^8^ sperm by using the extinction coefficient of NADPH as 6.22. The solution was incubated at 30°C for 5min and then read at 340nm on a spectrophotometer.

### Lactate concentration measurement

Sperm were disrupted by ultrasonic treatment in 0.9% NaCl and centrifuged at 14000g for 10 min. The supernatant was recovered and used to measure lactate concentration by enzymatic method measuring the NADH formed consequently to lactate oxidation by LDH as described by Gutmann and Wahlefeld [45] by spectrophotometry at 340nm. We used BPSE as a blank and a standard curve with increasing concentration of L(+) lactic acid was done before measurement of test samples.

### Citrate concentration measurement

The level of citrate in sperm was determined using commercial kit MAK057 (Sigma-aldrich, St Louis, MO, USA). Sperm was diluted in the citrate assay buffer and then centrifuged at 15000g for 10min to remove insoluble material. The colorimetric reaction of the supernatant was produced by the subsequent addition of 44μl Citrate buffer assay and 2μl of Citrate probe. Their action was started by the addition of 2μl of citrate enzyme mix and 2μl Citrate developer. The reaction mixture was vortexed and incubated at room temperature for 30min, protected from light. The absorbance in the supernatant was read at 570nm by spectrophotometer (Bio-Rad; Hercules, CA, USA). The absorbance values were compared to a standard curve in the concentration range of 0, 2, 4, 6, 8, and 10μl of the 1mM citrate standard, generating 0 (blank), 2, 4, 6, 8, and 10nmole/well standards.

### Western–Blotting

For western-blotting experiments, total proteins were extracted from frozen chicken sperm after thawing in lysis buffer (10mM Tris, 150mM NaCl, 1mM EGTA, 1mM EDTA, 100mM sodium fluoride, 4mM sodium pyrophosphate, 2mM sodium orthovanadate, 1% Triton X-100, 0.5% NP40 IGEPAL containing a protease inhibitor cocktail with EDTA). Cell lysates were centrifuged at 12000g for 30min at 4°C and protein concentration in each supernatant was determined by a colorimetric assay (Bio-Rad DC Protein Assay; Bio-Rad, Hercules, CA). The proteins were then separated by 10% SDS-PAGE (SDS Polyacrylamide Gel Electrophoresis) and transferred onto nitrocellulose membrane (Whatman Protran (Dassel, Germany)). Afterwards, the membranes were incubated in anti-AMPKα and anti-phospho-Thr172-AMPKα (62kDa) diluted in 5% BSA in TBS-Tween 0.1% (final dilution 1:1000) as primary antibodies overnight at 4°C. Finally, the membranes were further incubated for one hour in anti-rabbit IgG (H+L) (DyLight 680 Conjugate) secondary antibody (final dilution 1:2000). Bands were visualized by Infrared Fluorescence using the Odyssey Imaging System (LI-COR Inc. Biotechnology, Lincoln, NE, USA) and quantified by Odyssey infrared imaging system software (Application software, version 1.2). AMPKα was used as loading controls.

### Statistical analysis

Differences between treatments were analyzed by 1-way ANOVA and Bonferroni’s multiple comparison using GraphPad Prism version 5.0d for Mac (GraphPad Software, San Diego, CA). The minimum level of significance retained was P < 0.05.

## Results

### AMPK phosphorylation and modulation by its activators and inhibitor in frozen/thawed sperm

**Fig 1:**
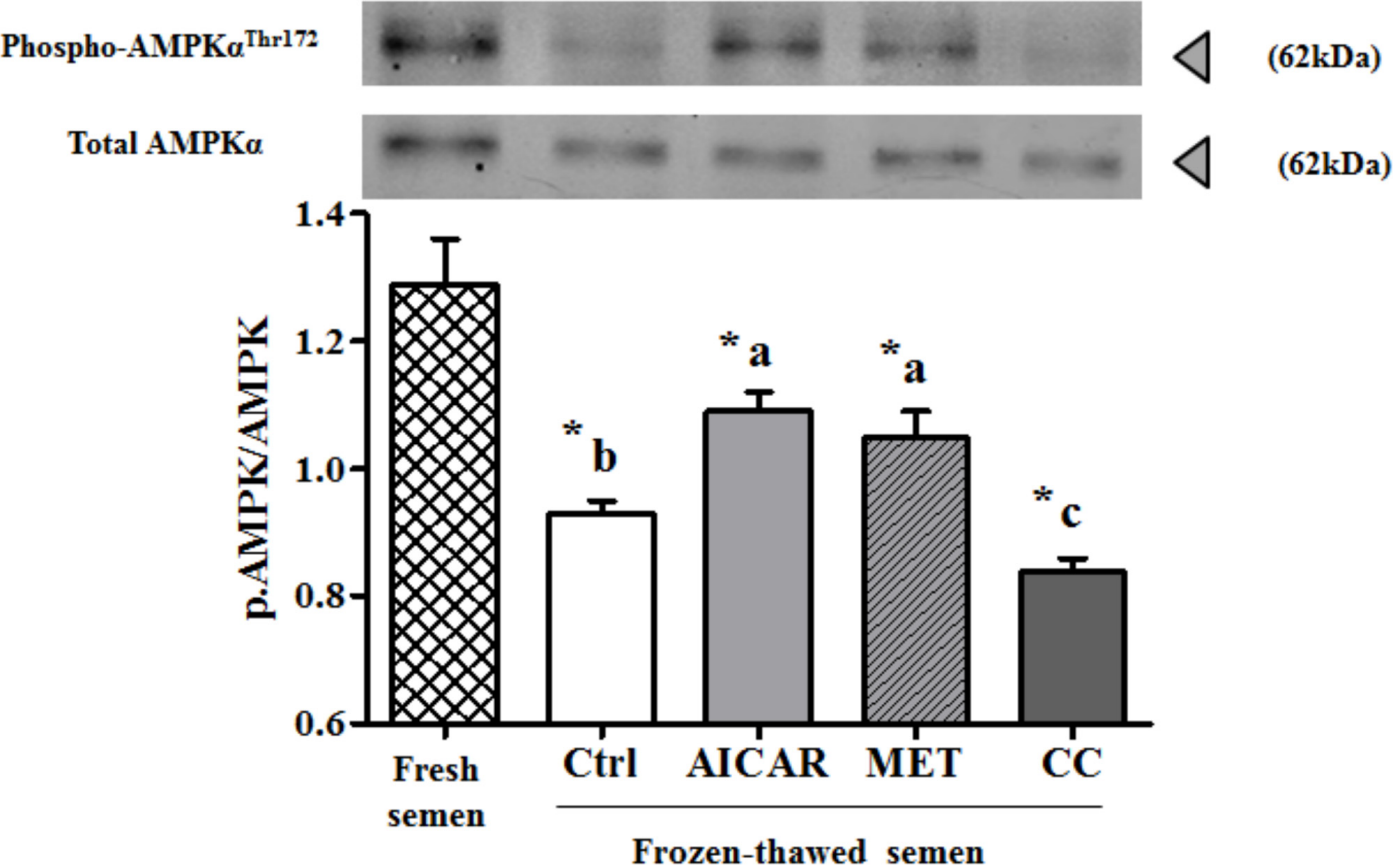
Modulators effects on AMPK phosphorylation in frozen-thawed chicken sperm. Sperm lysates were prepared and resolved by SDS-PAGE, transferred to nitrocellulose membrane, and probed with anti-phospho-Thr172-AMPKα and anti-AMPKα antibody. Bands for phospho-Thr^172^-AMPKα were detected at 62kDa (top lanes). Total AMPKα was used as loading control (62kDa) (bottom lanes) and the phosphorylated protein AMPKα (Thr172)/ total AMPKα ratio is shown at the bottom. Cryopreserved sperm were treated in the presence of an AMPK activator (2mM AICAR (in light gray) or 1mM MET (in diagonals)) or an AMPK inhibitor (5μM CC (in dark gray)) or Control (Ctrl, in white); Fresh semen (checkered pattern). Values represent means ± SEM from 6 different experiments (4 samples/experiment). Different superscripts indicate significant differences between Ctrl and treatments (AICAR, MET or CC) in frozen-thawed semen (^a,b,c^, P<0.05). Asterisks indicate statistically significant differences between fresh and frozen-thawed semen (*, P<0.05).

Western blot analyses using antibodies against phospho-Thr^172^-AMPKα and total AMPKα (as control) were performed on chicken sperm incubated in the absence or presence of 2mM AICAR, 1mM MET, or 5μM CC during freezing and thawing (Fig 1).

The AMPK phosphorylation was decreased by 30% after the freeze-thaw process. The decrease was higher in the presence of CC (35% decrease) but was only of 15.5% with AICAR and 18.6% with MET compared to fresh semen.

### Modulation of Reactive oxygen species (ROS) and lipid peroxidation (LPO) in frozen-thawed sperm by AICAR, MET or CC

**Fig 2:**
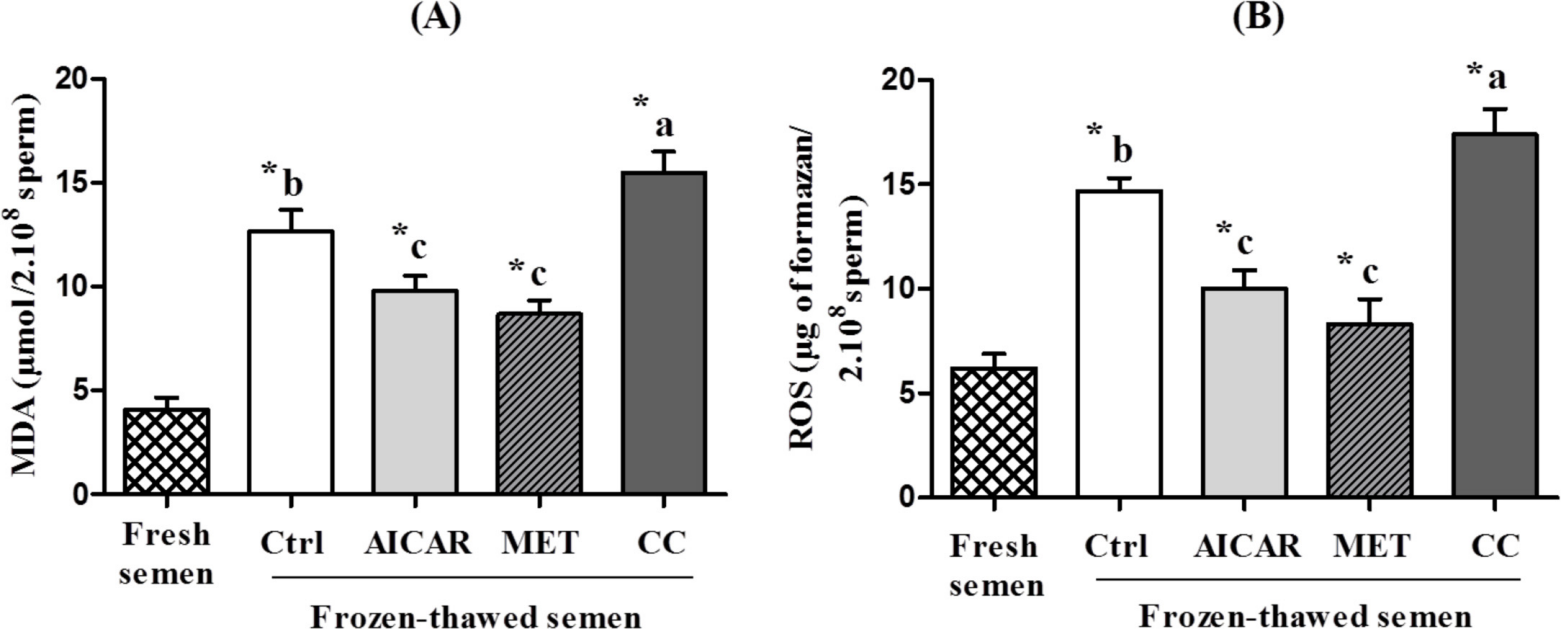
Effect of AMPK inhibitor CC and activators AICAR and MET on ROS and lipid peroxidation (MDA) in frozen-thawed chicken sperm. Sperm were treated in the presence of an AMPK activator (2mM AICAR (in light gray) or 1mM MET (in diagonals)) or an AMPK inhibitor (5µM CC (indark gray)) or Control (Ctrl, in white); Fresh semen (checkered pattern). **(A)** Lipid peroxidation (malondialdehyde), **(B)** Reactive oxygen species (ROS).Values represent means ± SEM from 6 different experiments (4 samples/experiment). Different superscripts indicate significant differences between Ctrl and treatments (AICAR, MET or CC) in frozen-thawed semen (^a,b,c^, P<0.01). Asterisks indicate statistically significant differences between fresh and frozen-thawed semen (*, P<0.001).

The freeze-thaw process strongly increased malondialdehyde (MDA) (+200%; Fig 2A) resulting from breakdown of fatty acids and taken as LPO index as well as ROS levels (+130%; Fig 2B). AMPK activators introduced before cryopreservation significantly lowered ROS levels of cryopreserved sperm: -32% for AICAR and -44% for MET compared to the control frozen-thawed without activator (Fig 2B). A decrease in MDA level was also observed with AICAR (-23%) and MET (-32%) compared to the control frozen-thawed without activator (Fig 2A). In contrast, CC increased both ROS (+18%) and MDA (+22%) levels after thawing relative to the control frozen-thawed without inhibitor.

### Activity of antioxidant enzymes in fresh and frozen thawed sperm

The antioxidant enzymes activity decreased greatly after cryopreservation with the exception of CAT (P<0.05, Table 1). The CAT activity was singular in frozen-thawed semen as MET significantly increased CAT activity while CAT activity remained statistically unchanged in the presence of AICAR or CC after thawing.

**Table 1:** Antioxidant enzymes activity of fresh and frozen-thawed chicken semen.

		Frozen-thawed semen			
Activity of antioxidant enzymes	Fresh semen	Ctrl	AICAR	MET	CC
SOD [U/2.10^8^ sperm]	26.6±2.5	9.7±0.8^b*^	16.0±1.6^a*^	19.2±1.2^a*^	6.2±1.3^c*^
CAT [U/2.10^8^ sperm]	6.2±0.6	5.1±0.5^b^	5.2±0.5^b^	7.7±0.6^a^	4.9±0.6^b^
GPx [mU/2.10^8^ sperm]	7.1±0.9	3.3±0.2^b*^	5.9±0.9^a^	5.2±1.1^a^	3.3±0.2^b*^
GR [mU /2.10^8^ sperm]	6.5±0.5	4.5±0.1^b*^	5.4±0.2^a*^	5.6±0.3^a^	4.5±0.2^b*^

Sperm were treated with 2mM AICAR, 1mM MET or 5µM CC. Values represent means ± SEM from 6 different experiments (4 samples/experiment).

Different superscripts in lines indicate significant differences between control (Ctrl) and treatments in frozen-thawed semen (^a,b,c^, P<0.05). Asterisks in lines indicate statistically significant differences between fresh and frozen-thawed semen (*, P<0.05).

The decreases in SOD, GPx and GR activities following cryopreservation were greatly attenuated in the presence of an activator, AICAR or MET, in the freezing medium. These AMPK activators led to almost full restoration of the initial levels of GPx and GR activities and to partial restoration of SOD (P<0.05). The presence of CC decreased SOD activity but did not affect GSH and GPx activities measured after thawing (Table 1).

### Sperm metabolism indices

#### Mitochondrial membrane potential and ATP

**Fig 3:**
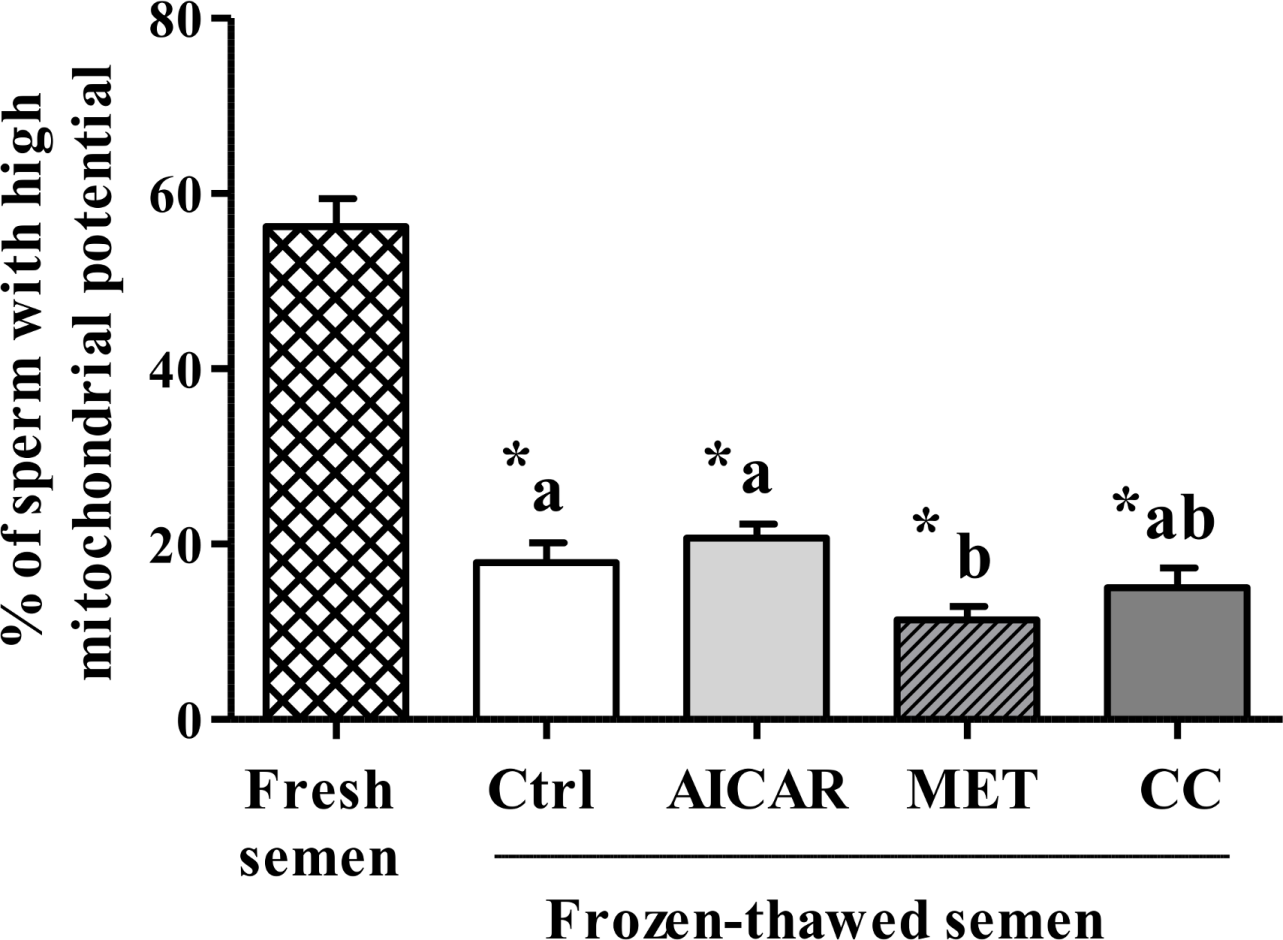
Effect of AMPK inhibitor CC and activators AICAR and MET in mitochondrial membrane potential of frozen-thawed sperm. Sperm were treated in the presence of an AMPK activator (2mM AICAR (in light gray) or 1mM MET (in diagonals)) or an AMPK inhibitor (5µM CC (in dark gray)) or Control (Ctrl, in white); Fresh semen (checkered pattern).Values represent means ± SEM from 6 different experiments (4 samples/experiment). Different superscripts indicate significant differences between Ctrl and treatments (AICAR, MET or CC) in frozen-thawed semen (^a,b^, P<0.05). Asterisks indicate statistically significant differences between fresh and frozen-thawed semen (*, P<0.001).

**Fig 4:**
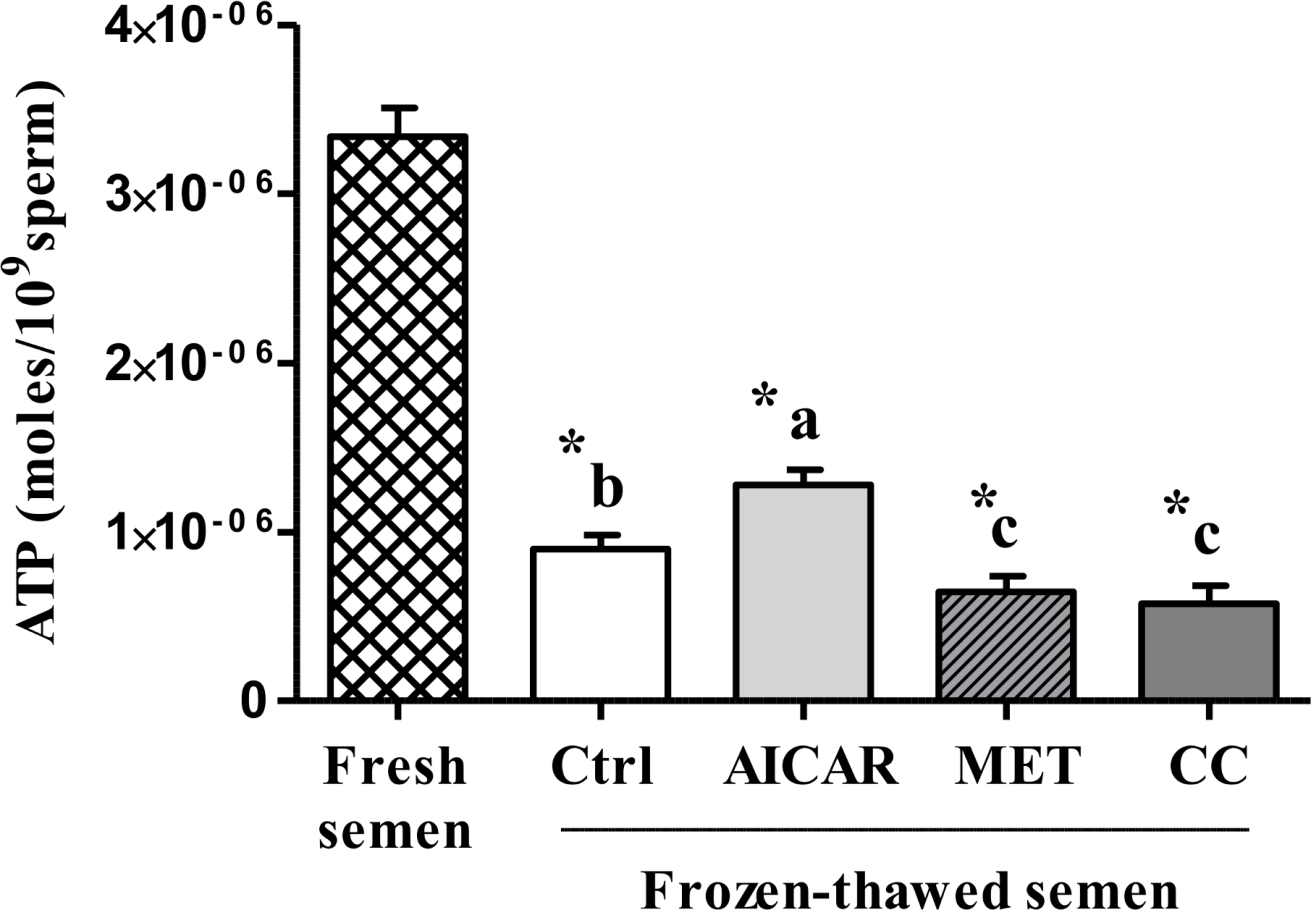
Effect of AMPK inhibitor CC and activators AICAR and MET on ATP concentration in frozen-thawed sperm. Sperm were treated in the presence of an AMPK activator (2mM AICAR (in light gray) or 1mM MET (in diagonals)) or an AMPK inhibitor (5μM CC (in dark gray)) or Control (Ctrl, in white); Fresh semen (checkered pattern). Values represent means ± SEM from 6 different experiments (4 samples/experiment). Different superscripts indicate significant differences between Ctrl and treatments (AICAR, MET or CC) in frozen-thawed semen (^a,b,c^, P<0.05). Asterisks indicate statistically significant differences between fresh and frozen-thawed semen (*, P<0.001).

Cryopreservation induced a 68% loss of mitochondrial potential as measured with the JC1 dye and 70% decrease of ATP content (Fig 3). The presence of MET in the freezing medium led to a 36% decrease of mitochondrial potential compared to control whereas AICAR or CC had no effect. AICAR significantly increased the ATP concentration of the cryopreserved sperm but MET and CC significantly decreased it (Fig 4).

#### Lactate production

**Fig 5:**
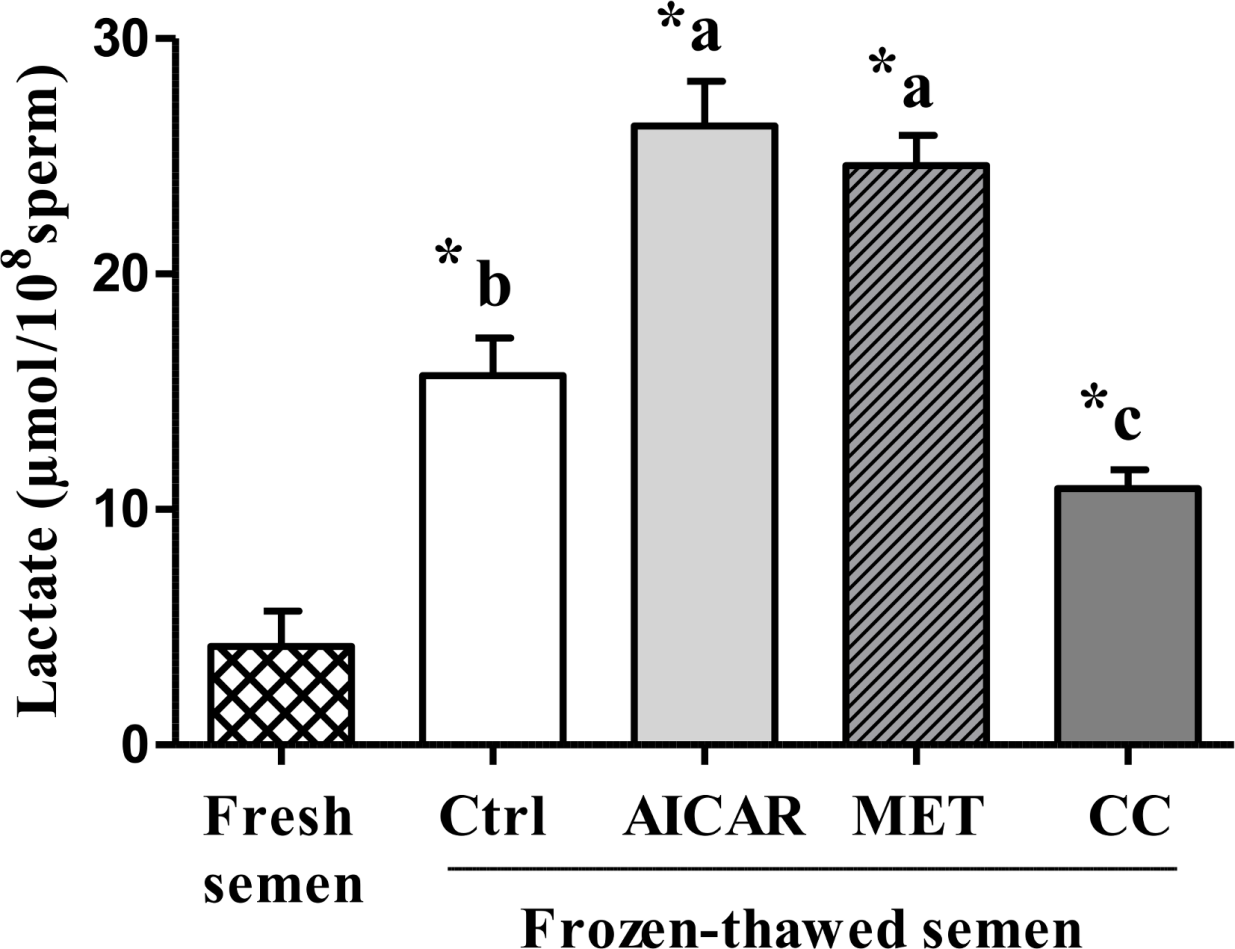
Effect of AMPK modulators (AICAR, MET and CC) on frozen-thawed sperm lactate production. Sperm were treated in the presence of an AMPK activator (2mM AICAR (in light gray) or 1mM MET (in diagonals)) or an AMPK inhibitor (5μM CC (in dark gray)) or Control (Ctrl, in white); Fresh semen (checkered pattern). Values represent means ± SEM from 6 different experiments (4 samples/experiment). Different superscripts indicate significant differences between Ctrl and treatments (AICAR, MET or CC) in frozen-thawed semen (^a,b,c^, P<0.01). Asterisks indicate statistically significant differences between fresh and frozen-thawed semen (*, P<0.001).

Lactate level in frozen-thawed sperm significantly increased compared to fresh sperm (+200%; Fig 5). Moreover, addition of an AMPK activator before freezing led to an additional significant increase in lactate level (+68% with AICAR and +57% with MET relative to the control without activator). In contrast, addition of AMPK inhibitor CC significantly inhibited the lactate level by 30% after thawing relative to the control frozen-thawed without inhibitor (Fig 5).

#### Citrate production

**Fig 6:**
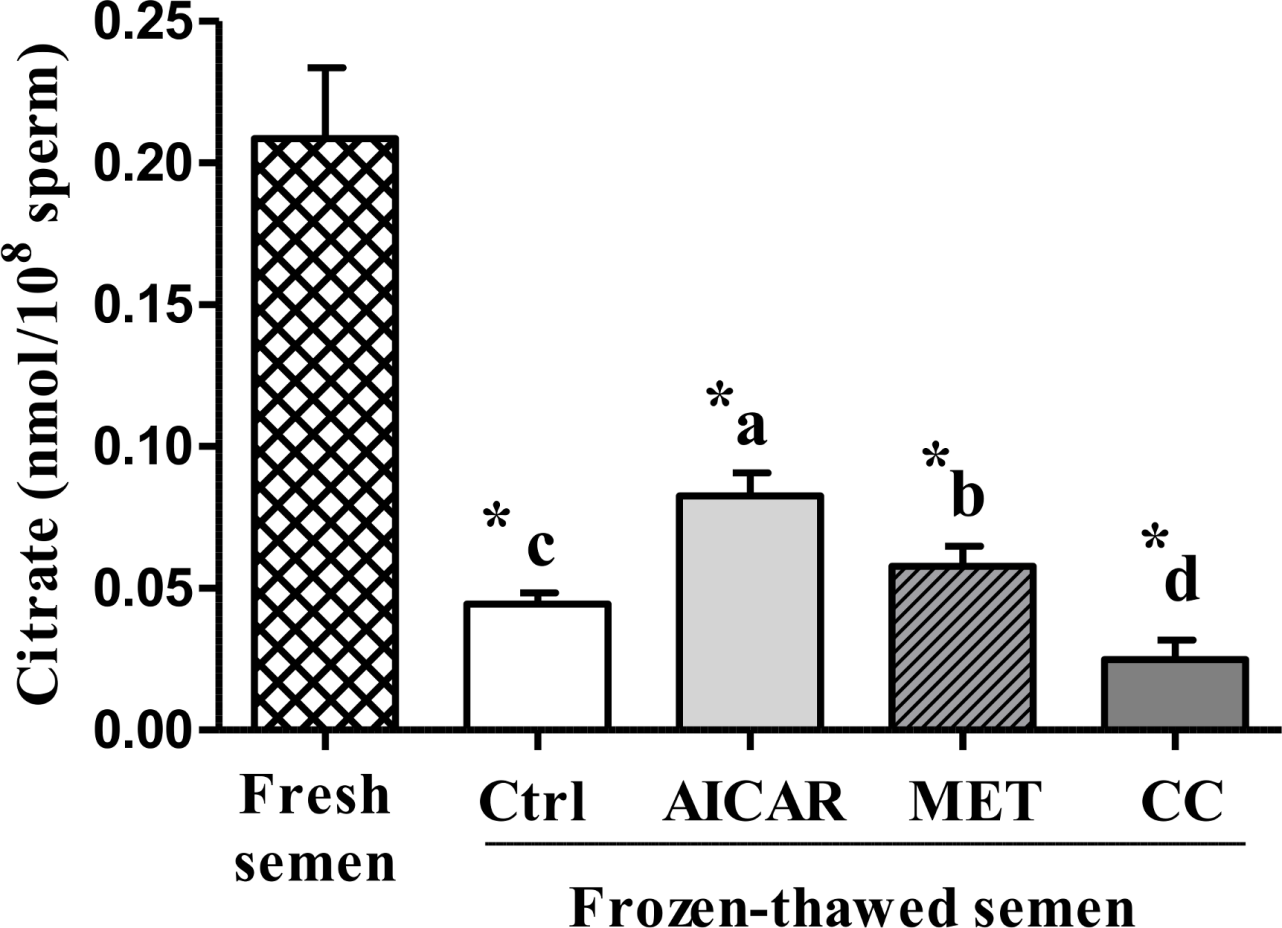
Effect of AMPK modulators (AICAR, MET and CC) on frozen-thawed sperm citrate level. Sperm were treated in the presence of an AMPK activator (2mM AICAR (in light gray) or 1mM MET (in diagonals)) or an AMPK inhibitor (5μM CC (in dark gray)) or Control (Ctrl, in white); Fresh semen (checkered pattern). Values represent means ± SEM from 6 different experiments (4 samples/experiment). Different superscripts indicate significant differences between Ctrl and treatments (AICAR, MET or CC) in frozen-thawed semen (^a,b,c,d^, P<0.05). Asterisks indicate statistically significant differences between fresh and frozen-thawed semen (*, P<0.001).

The citrate level in frozen-thawed sperm dramatically decreased compared to fresh sperm (-79%; Fig 6). Moreover, addition of AMPK activator before freezing-thawing sperm led to significant increases in citrate levels (+85% with AICAR and +30% with MET compared to the frozen-thawed control). In contrast, addition of CC further lowered the citrate level (-44%) after thawing (Fig 6).

### Sperm biological functions (motility parameters, acrosome reaction and viability)

The values presented in Table 2 show a detrimental effect of the cryopreservation process on sperm motility parameters, acrosome reaction and viability. Freezing and thawing led to significant decreases of sperm motility (-37%), VAP, and VSL. The percentage of sperm able to undergo a successful acrosome reaction was dramatically reduced after cryopreservation (-74%). Sperm viability was decreased from 86% (before cryopreservation) to 54% (after cryopreservation and thawing).

**Table 2:** Effect of AICAR, MET or CC treatment on fresh sperm and following cryopreservation.

		Frozen-thawed semen			
Motility parameters	Fresh semen	Ctrl	AICAR	MET	CC
Motile sperm (%)	76.2±3.0	47.8±2.5^b*^	62.2±1.4^a*^	58.8±1.6^a*^	37.6±2.5^c*^
Rapid sperm (%)	43.6±3.0	23.5±2.7^b*^	35.5±4.4^a*^	35.0±4.2^a*^	18.8±1.4^c*^
Progressive (%)	26.4±1.2	12.0±1.1^b*^	22.3±3.1^a^	20.2±2,9 ^a*^	10.0±0,9^b*^
VAP (μm\s)	65.8±1.9	54.7±1.9^b*^	61,1±2.9^a^	62.5±3.6^a^	53,0±2.2^b*^
VSL (μm\s)	53.0±1.4	43.6±2.2^a*^	49.7±3.6^a^	50.0±4.7^a^	41.8±2,1^a*^
VCL (μm\s)	104.1±3.3	101.1±2.5^ab^	104.7±3.1^ab^	105.1±2.7^a^	98,1±2.4^b^
STR (μm\s)	74.4±0.6	75.0±1.6^a^	75.6±2.6^a^	74.8±1.4^a^	74.8±0,5^a^
LIN (μm\s)	45.2±0.7	43.3±2.0^a^	44.5±2.5^a^	44.0±2.4^a^	42,0±1,4^a*^
AR (%)	36.7±1.0	9.4±0.5^b*^	13.4±0.7^a*^	12.4±0.7^a*^	6.4±0.4^c*^
Viable sperm (%)	86.2±3.8	53.8±0.9^b*^	56.2±1.5^ab*^	57.8±1.3^a*^	53.6±1.5^b*^

Sperm were treated with 2mM AICAR, 1mM MET or 5μM CC. The sperm motility parameters including motile sperm (%), rapid sperm (%), progressive (%), path velocity (VAP, in μm/s), progressive velocity (VSL, in μm/s), curvilinear velocity (VCL, in μm/s), straightness coefficient (STR, in %), linearity (LIN, in %) were evaluated by the computer-assisted sperm analysis (CASA) system. Sperm viability (%) was assessed using SYBR-14/PI. Acrosome reaction (AR, %) was assessed using a FITC-PNA staining. Values represent means ± SEM from 6 different experiments (4 samples/experiment).

Different superscripts in lines indicate significant differences between control (Ctrl) and treatments in frozen-thawed semen (^a,b,c^, P<0.05). Asterisks in lines indicate statistically significant differences between fresh and frozen-thawed semen (*, P<0.05).

#### Effects of AMPK modulators on sperm motility parameters after thawing

The addition of an activator, either AICAR or MET, significantly increased cryopreserved sperm motility parameters: up to 30% of motile, 86% of progressive and 51% of rapid cells with AICAR and up to 23% of motile, 68% of progressive and 49% of rapid cells with MET (Table 2). Conversely, treatment of chicken sperm with CC significantly decreased motile (-21%) and rapid cells (-20%), but did not significantly affect the percentage of progressive sperm (Table 2). The VAP values were also positively affected by the presence of the AMPK activators but not by the CC inhibitor and the other motility parameters (VSL, VCL, STR, and LIN) did not change in the presence of AMPK modulators compared to control after thawing (Table 2).

#### Effects of AMPK modulators on the ability of cryopreserved sperm to undergo the acrosome reaction

AR was severely affected by cryopreservation, but partially restored in the presence of AICAR (+43%) or MET (+32%) relative to control frozen-thawed sperm. In contrast, it was even more decreased (-32% relative to control frozen-thawed sperm) in the presence of CC (P<0.05, Table 2).

#### Effects of AMPK modulators on cryopreserved sperm viability

The sperm viability rates were not affected in presence of AICAR or CC (mean 53–56%). In contrast MET significantly increased sperm viability by 8% compared to the control (P<0.05, Table 2).

## Discussion

The present study is the first showing a positive effect of AMPK activators on the capacity of mature sperm to restore their biological functions after cryopreservation. It is also the first evaluating the role of AMPK on the quality of bird cryopreserved semen. Our data indicate that AMPK activation plays a key role by contributing to restore antioxidant enzymes system, removing ROS, limiting lipid peroxidation (LPO), and thus allowing an increase in motility, acrosome reaction and viability as well as numerous other parameters of sperm metabolism and functions.

Cryopreservation is well-known for its deleterious effects on most cells and especially for sperm that are highly differentiated cells with inactive genome. Facing such stressful conditions, sperm require energy and defense or reparation systems to preserve their essential functions. The cellular defense mechanisms such as the anti-oxidant enzymes are expected to be highly solicited and the metabolic functions need to adapt. AMPK signaling was thus expected to play an important role. In our study on chicken sperm freezing, we show that a/ the capacity of stimulation of regulating kinases such as AMPK is affected after cryopreservation (Fig 1), b/ the ROS and LPO productions dramatically increase (Fig 2), c/ the ability of sperm to activate the aerobic metabolic pathways involving mitochondrial functions are severely decreased (Figs 3 and 6), and d/ the ATP production is dramatically altered after cryopreservation (Fig 4). Through the strong increase in lactate production induced by cryopreservation (Fig 5), our results clearly suggest that the anaerobic glycolysis pathway is much more solicited after cryopreservation for maintaining basal ATP production. This pathway would be much less efficient than the aerobic pathway and many glycolysis enzymes would be limiting [46]. Finally, the intracellular antioxidant enzymes acting in parallel with oxidative phosphorylations are also altered (Table 1). Part of our results in chicken sperm confirm previous studies in other species on ROS [19] and MDA productions as well as CAT, GPx and SOD levels [26], mitochondrial potential [47] or ATP [6], but most of them are original whatever the species and piece together numerous data from mammals and birds.

Because AMPK is a key kinase involved in stressful conditions, our hypothesis was that its stimulation by different activators would increase motility of cryopreserved sperm as suggested by a previous study on epididymal mouse sperm [48], as well as AR and different metabolic functions. AR was studied with particular attention as it has been previously shown that chicken sperm cryopreserved in 11% glycerol, as it is the case in the present work, lose most of their AR ability immediately after their initial contact with glycerol [49].

Our observation that the direct (AICAR) and the indirect (MET) AMPK activators increase AMPK phosphorylation together with motility parameters and AR capability of cryopreserved sperm and that the AMPK inhibitor (CC) promotes the opposite effects confirms our hypothesis. These results differed from those obtained with stallion sperm where AMPK modulators (AICAR, MET and CC) did not affect sperm viability and motility after cryopreservation [50]. However, in addition to the use of highly different doses of MET and CC in the two studies, the work on stallion sperm of Cordova *et al*. 2014 [50] used a very specific hypo-metabolic medium of sperm storage with restricted access to energetic substrate that greatly limits the potential comparisons with our study. In another study (Martin-Hidalgo D*et al*. 2013 [51]), a lack of effect of CC was observed in boar sperm stored *in vitro* but the conditions of liquid storage and the much higher CC concentration used in this study do not permit efficient comparisons with our results. More surprisingly, but in accordance with a previous study on epididymal mice sperm [48], MET showed a low but significant positive effect on sperm viability. This is not directly linked to AMPK regulation since AICAR did not exhibit the same effect.

In order to explain the positive action of AMPK stimulation on frozen sperm functions, we investigated the effects of AMPK activators/inhibitor on ROS and LPO formation. Free radicals are known as regulatory mediators in signaling processes, as well as in cell proliferation, differentiation, and migration [52]. However, at high concentrations, free radicals are hazardous for living organisms and damage all major cellular constituents through oxidative stress by the excessive production of ROS [53]. Previous studies have provided evidence that AICAR suppresses ROS production in endothelial cells through AMPK activation [14] and CC showed an opposite effect [54]. Similarly, MET was shown to exert an anti-inflammatory effect on non-alcoholic steato-hepatosis mice through both AMPK-dependent and AMPK-independent pathways [55] by impeding depletion in GPx, SOD, and catalase, and by decreasing ROS and MDA [10]. These findings support the idea that MET and AICAR promote antioxidative responses through AMPK phosphorylation whereas CC promotes a pro-oxidative effect through inhibition of AMPK phosphorylation. Our results on cryopreserved chicken sperm support the same hypothesis since ROS and MDA where reduced by AICAR and MET but increased by CC. However, MET could also directly reduce ROS production via inhibition of complex I. Indeed, the inhibition of complex I by MET is known to reduce the number of electrons entering the electron transport chain, thus blocking NADH oxidation by complex I [56, 57], and therefore reducing ROS production by both complex I and III [58].

The reduction of the sperm antioxidant enzyme activities due to cryopreservation largely abolished their ability to maintain semen quality in our study. SOD, GPx, GR, and CAT are the major enzymes involved in the removal and detoxification of ROS and have been described as functioning as defense mechanisms against LPO in semen of human and numerous animal species [21, 22]. SOD is considered as the main element of initial antioxidative defense in the semen [59]. SOD catalyzes dismutation of the superoxide anion into molecular oxygen and hydrogen peroxide which, in turn, is removed by GPx and catalase [60]: O_2_^•−^ + O_2_^•−^+ 2H^+^→H_2_O_2_ + O_2_. GPx catalyzes the reduction reaction, not only of H_2_O_2_, but also of organic peroxides, especially lipid peroxides with reduced glutathione (GSH): 2GPx + H_2_O_2_→ GSSG + 2H_2_O. Glutathione disulfide (GSSG), which is formed in this reaction, is deleterious to cells by affecting their redox potential, but it is reduced into GSH by glutathione reductase (GR) via oxidation of NADPH: GSSG + NADPH + H^+^→ NADP^+^ + 2GSH. CAT detoxifies hydrogen peroxide into oxygen and water and has no electron donor requirement: 2H_2_O_2_→2H_2_O + O_2_. Avian semen contains two SOD forms (mitochondrial Mn-SOD and cytoplasmic Cu, Zn-SOD). Mn-SOD is the main form in avian sperm (57% of total SOD in chicken). By contrast, Cu, Zn-SOD is the only form present in seminal plasma [61]. GPx is mainly present in its Se-dependent form in chicken (77.7% of total) [61]. Recently, CAT activity was detected in chicken semen [26] but the present study is the first reporting GR activity in chicken sperm.

We have shown that the addition of MET or AICAR to sperm before cryopreservation significantly increased GR, GPx, and SOD activities in frozen-thawed sperm and decreased at the same time LPO and ROS, and that semen quality was improved. In contrast, CC addition decreased SOD activity and increased LPO and ROS with negative effects on sperm quality. Thus, our data indicate that treatment with AMPK activators (MET or AICAR) prevented oxidative burst by increasing the activity of the anti-oxidative enzymes involved in frozen thawed chicken sperm metabolism. Loss of antioxidant enzymes after cryopreservation has been reported to be, at least partly, due to altered membrane integrity of unviable sperm [23]. However, in our study, the increase in anti-oxidant enzymes after cryopreservation could mainly be related to a recovery of the living cells enzymes activity since the stimulation is equivalent with AICAR and MET despite a small positive effect of MET (but not AICAR) on the percentage of viable sperm. On the other hand, MET (and not AICAR), stimulated CAT activity through an unknown mechanism, probably unrelated to AMPK as found for other cell types [62]. Previous studies have shown that AMPK activation could directly reduce ROS production by restoring Mn-SOD levels in endothelial progenitor cells [63]. In this study, we suggest that AICAR and MET increase the antioxidant enzymes SOD, GR, and GPx activities through AMPK activation. However, the precise mechanism of the antioxidant effect of AMPK activation is not completely understood. Quite recently, Canto C *et al*. 2009 have reported that MET or AICAR activation of AMPK in muscle triggers an increase in the NAD+/NADH ratio, which activates Sirtuin 1 (SIRT1) [64]. AMPK also induces the phosphorylation of peroxisome proliferator activated receptor γ coactivator (PGC-1α) and primes it for subsequent deacetylation by SIRT1. PGC-1α and SIRT1 are present in mitochondria and have a role in regulation of mitochondrial biogenesis [65]. We thus suggest that the impact of AMPK on SIRT1 and on PGC-1α could then modulate the mitochondrial function, increasing the mitochondrial antioxidant enzymes MnSOD, GPx, GR and decreasing ROS.

The AMPK inhibitor CC also acts directly on AMPK phosphorylation, and has been shown to abolish the effects of hypoxic preconditioning in rat embryonic-heart derived H9c2 cell by decreasing SOD activity and increasing ROS and MDA levels [66]. In our study, the inhibitory effect of CC on AMPK related functions was not complete since CC affected all expected functions with the exception of GPx and GR. Since CC had to be diluted in DMSO that is toxic to sperm, only CC concentrations far lower than those of AICAR and MET could be studied. Therefore, AMPK might not have been completely inhibited by the low CC level used. Overall, our results suggest that AMPK activation in frozen thawed chicken sperm could, at least partially, restore sperm antioxidative activities.

In order to explain the role of AMPK in energy balance and subsequent changes in frozen sperm functions, we investigated the effects of AMPK activators/inhibitors on different metabolic indicators such as mitochondrial activity, ATP, lactate, and citrate levels. Mitochondria are major cell sources of oxidative energy through their production of ATP via the electron transport chain (ETC). The ETC itself is made up of subunits that are encoded by the mitochondrial DNA (except complex II) [67]. The mitochondrial potential was shown to be severely affected by cryopreservation in our study as well as ATP production. However, the relationship between ATP level and mitochondrial activity was not clear as ATP production was modulated after cryopreservation by AMPK activators and inhibitor but not the mitochondrial potential that was still very low whatever the treatment. Mitochondria are key organelles for the success of aerobic metabolism that are severely affected by the freeze-thaw process. Consequently, the anaerobic glycolysis would be much more solicited after cryopreservation for maintaining basal ATP production as shown by the large increase in lactate production and the opposite decrease in citrate level after cryopreservation. Moreover, AICAR and MET increased both lactate and citrate production after cryopreservation while CC inhibited them. AMPK activation would thus improve the metabolism status through glucose uptake and/or stimulating glycolysis, inducing increased ATP concentration and the production of the lactate end product of glycolysis [68, 69]. But AMP (and AICAR) may also act directly on glycolysis, leading to the suggestion of two routes of action: 1) AMPK-dependent mechanism: the AMPK activation indirectly stimulates the 6-phosphofructo-1-kinase (PFK-1), an allosteric enzyme that occupies the key regulatory position for glycolysis [70], by phosphorylating and activating 6-phosphofructo-2-kinase, the enzyme that synthesizes fructose 2,6-bisphosphate, a potent PFK-1 stimulator [71]; 2) Direct AMP effects: AICAR increases ZMP, which mimics AMP [72], the latter being an activator of PFK1. This may also help to explain some of the metformin effects since Metformin is known to increase AMP in hepatocytes [73].

Citrate is a key TCA cycle intermediate formed by addition of oxaloacetate to the acetyl group of acetyl-CoA to provide ATP for the electron transport chains [73]. The fact that citrate production can still be modified by the AMPK activators/inhibitor after cryopreservation indicates that frozen-thawed sperm still retain a small potential for TCA cycle mobilization despite mitochondrial damages.

Thus, midpiece damages caused by freezing and thawing procedures would lead to significant reduction in TCA cycle/oxidative phosphorylation activity and to large losses of ATP. In reaction, with a major role in regulating cellular energy balance, AMPK stimulates anaerobic glycolysis and the TCA cycle to synthesize ATP. The MET action would be singular since it might stimulate the activation of TCA cycle to produce ATP but not the electron transport chain since MET inhibits Complex I. Thus, we suggest that in conditions of oxidative stress caused by cryopreservation, when oxidative phosphorylation is impaired, the contribution of glycolysis to the synthesis of ATP is required to maintain cell functions and viability.

In conclusion, the results of the present paper demonstrate that chicken sperm cryopreservation in the presence of AMPK activators not only improves the post-thaw motility and AR of healthy sperm by restoring ATP level and antioxidant system (SOD, GPx, and GR), but also reduces oxidative stress and lipid peroxidation. This is the first assessment of the effects of MET, AICAR, or CC on frozen-thawed chicken sperm through their influence on AMPK activity to reduce the causes of cryopreservation damages in avian sperm. Such data will most certainly be helpful to develop and improve semen handling and storage techniques in the near future.
